# A novel fully guided technique for botulinum toxin injection in lateral pterygoid muscle using muscle segmentation for TMJ disc displacement with reduction: a randomized controlled trial

**DOI:** 10.1186/s12903-025-06372-w

**Published:** 2025-06-21

**Authors:** Nourhan Raafa, Lydia Melek, Hesham Zoheir, Eman Mansour, Aya Sakr

**Affiliations:** 1 Department of Oral and Maxillofacial Surgery, Faculty of Dentistry, Alexandria University, Alexandria, Egypt; 2 Consultant of Diagnostic and Interventional Radiology, Faculty of Medicine, Alexandria University, Alexandria, Egypt; 3 Department of Rheumatology, Rehabilitation and Physical Medicine, Faculty of Medicine, Alexandria University, Alexandria, Egypt

**Keywords:** Disc displacement with reduction, Botulinum toxin, Lateral pterygoid muscle, Muscle segmentation, Electromyography

## Abstract

**Objective:**

To evaluate the effectiveness of fully guided botulinum toxin (BTX) injection in lateral pterygoid muscle (LPM) using muscle segmentation technique and compare it with electromyography (EMG) for management of symptomatic disc displacement with reduction (DDWR).

**Materials and methods:**

This prospective randomized controlled trial (RCT) included 20 patients suffering from DDWR. Patients were randomly allocated to two groups receiving BTX injection in LPM using fully guided technique of muscle segmentation for LPM in group I (study) while using EMG in group II (control). Evaluation was done for disc position after 3 months and for maximum interincisal opening (MIO), temporomandibular joint (TMJ) and LPM tenderness, and clicking after 1,3 and 6 months.

**Results:**

Maximum interincisal opening showed reduction at 1-month follow-up, followed by significant improvement in both groups. Significant disappearance of clicking, reduction in LPM and TMJ tenderness and disc position reduction were detected in both groups. However, the difference between both groups was not statistically significant except in LPM tenderness, there was a statistically significant difference in favor of group I at 3-and 6- month follow-up.

**Conclusion:**

The findings suggest that the fully guided technique using muscle segmentation is a viable, cost-effective and reproducible alternative to EMG for BTX injection in LPM.

**Clinical relevance:**

The fully guided technique by muscle segmentation in LPM is as effective as EMG, providing 3D virtual augmented environment of the muscle with its surrounding skeletal and dental structures.

**Trial registration:**

This prospective RCT has been retrospectively registered at Clinical Trials.gov with identification number: NCT06633445, 2024–10-01.

## Introduction

Temporomandibular disorders (TMDs) are one of the most prevalent musculoskeletal conditions resulting in pain and dysfunction that adversely affect quality of life of those affected [1].Temporomandibular disorder is a collective group of disorders affecting muscles of mastication, temporomandibular joint (TMJ) and its associated structures, encompassing several types such as arthralgia, disc displacement, dislocation, degenerative joint disease and myofascial pain [1–3].Disc displacement is considered the most frequent cause for TMJ sounds [4]. It has been classified by the Diagnostic Criteria of Temporomandibular Disorders(DC/TMD) into 4 types: disc displacement with reduction (DDWR), DDWR with intermittent locking, disc displacement without reduction (DDWoR) with limited opening and DDWoR without limited opening, with DDWR with or without intermittent locking being the most frequent one [1, 5, 6]. In DDWR, the articular disc is most commonly displaced anteriorly to the condyle in closed mouth and regains its position upon opening the mouth [1, 6]. Affected patients usually seek treatment when symptoms such as clicking and pain are persistent and interfere with daily function, therefore, the appropriate treatment plan for these patients should be considered to avoid any potential harms [7–9]. Management of DDWR starts with conservative modalities such as physical therapy, biofeedback therapy, pharmacological therapy; however, joint clicking, being the main complaint, usually does not improve after these conservative modalities [7–12]. Therefore, arthrocentesis, TMJ arthroscopy and botulinum toxin (BTX) injection in the lateral pterygoid muscle (LPM) are another minimally invasive procedures for patients resistant to previous modalities [5, 9, 13, 14]. Choice of the appropriate treatment for each patient should be considered to avoid any harm.

Botulinum toxin is a biologic toxin produced from fermentation process of Clostridium botulinum bacteria. This toxin affects presynaptic neuron in neuromuscular junction resulting in muscle relaxation by preventing acetylcholine release [15, 16]. There are several types of BTX, with type A (BTX-A) being the most widely used due to its safety, duration and effectiveness [15]. In addition, BTX shows analgesic and anti-inflammatory effects on managing some chronic pain conditions that are unresponsive to traditional analgesics [17, 18].

Several studies have suggested injecting BTX in LPM for management of DDWR, based on the theory that LPM hyperactivity displaces the articular disc anteriorly and counteracts the action of elastic retrodiscal tissues. [5, 7, 9, 11]. Lateral pterygoid muscle is difficult to access and target due to the deep location and the presence of multiple vital structures as well as interindividual variations [19–21]. Blind LPM injection is sometimes accompanied with dysphagia and dysphonia due to diffusion of BTX to surrounding structures as pharyngeal muscles and soft palate, therefore, guided BTX injection in LPM is mandatory and different guidance options were introduced [20–22].

Recently, virtual planning together with augmented reality facilitate creation of 3D virtual anatomical environment of multiple body regions. These technologies have been widely used in oral and maxillofacial procedures specially in implantology and orthognathic surgeries [23, 24].

A novel technique is introduced in the current study in which muscle segmentation for LPM is done to create augmented environment of the muscle and its surrounding structures to facilitate virtual planning for guided BTX injection, thus precluding the need for conventional confirmation with electromyography (EMG).

This clinical trial aims to evaluate the effectiveness of fully guided BTX injection using muscle segmentation technique for LPM compared to EMG, the most commonly used guided technique, for management of DDWR patients.

## Materials and methods

**Fig.1:**
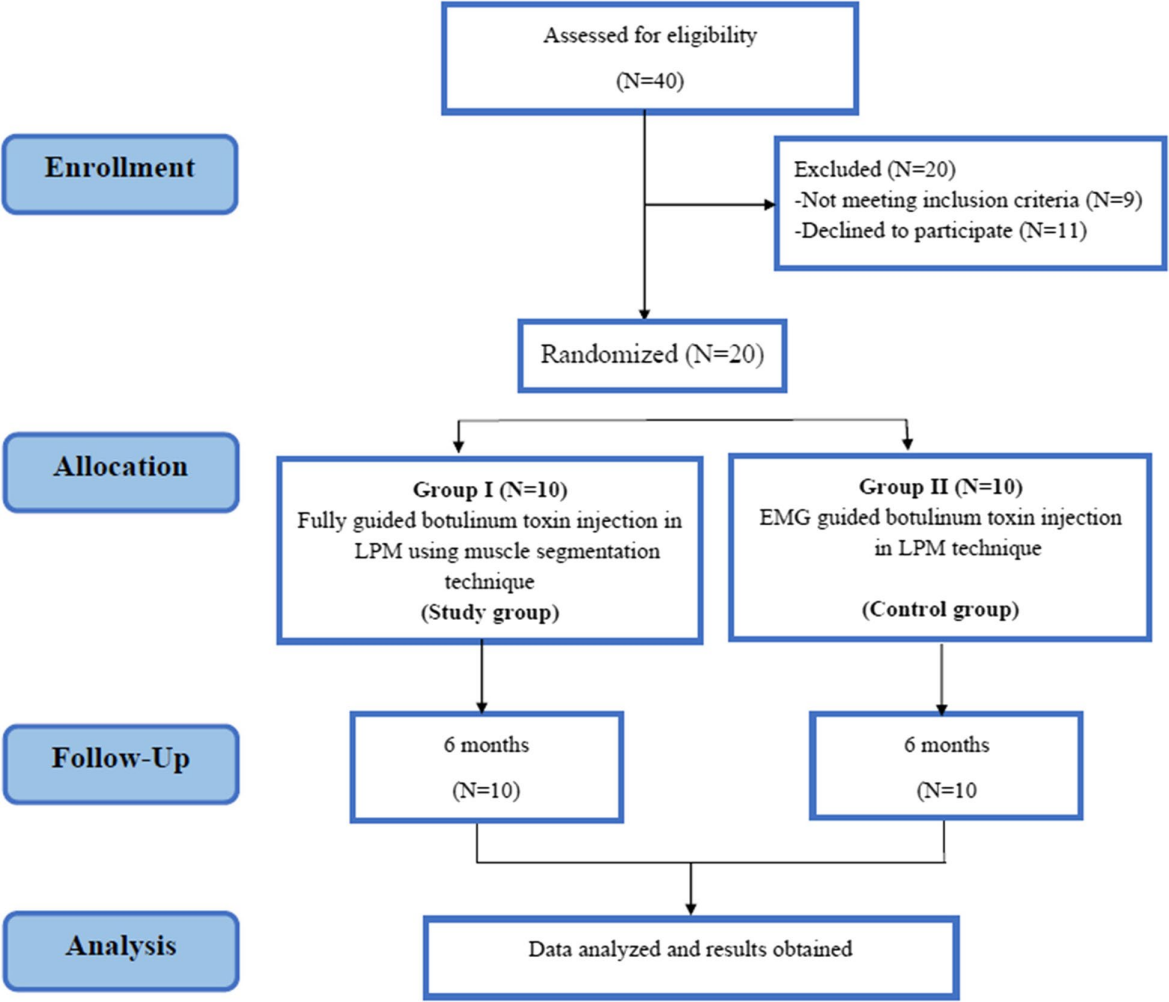
CONSORT Flow chart

Informed consent from all 20 patients were obtained before participating in the trial. This study followed the CONSORT criteria and Helsinki principles [25, 26]. (Fig. 1).

### Study design

This prospective, double blinded randomized controlled clinical trial has been retrospectively registered at Clinical Trials.gov with identification number: NCT06633445, 2024–10-01 and approved by the Research Ethics Committee, Faculty of Dentistry, Alexandria University, Egypt (IRB No.001056—IORG 0008839; ethics committee number:0837–01/2024; date of approval: 2024–01–16).

### Inclusion and exclusion criteria

Twenty patients from both sexes with pain and clicking in TMJ were enrolled in the study after fulfilling the inclusion criteria which were:

1. Angle class I maxillomandibular relation.
2. 20 to 45 years old age range.
3. Diagnosed with DDWR according to DC/TMD and confirmed with MRI.

While the exclusion criteria were:

1. Degenerative joint disease, musculoskeletal, bleeding disorders, neurological disorders.
2. Pregnant and lactating women.
3. History of surgical TMJ intervention.
4. Botox hypersensitivity.
5. Posteriorly edentulous saddles or anterior open bite.
6. Patient contraindicated to MRI examination.

Patients fulfilling these criteria were enrolled after meticulous clinical examination according to DC/TMD and MRI examination of TMJ.

## Intervention

### A) For both groups, preoperative measurements for all enrolled patients were obtained

#### Maximum interincisal opening

Unassisted maximum interincisal opening (MIO) was obtained by measuring the vertical distance between the incisal edges of maxillary and mandibular central incisors in millimeters.

#### TMJ clicking

TMJ clicking was recorded as present or absent. Assessment was done by bilateral palpation by the index fingers to the lateral pole of the condyle in front of ear tragus while asking the patient to open and close several times.

#### LPM and TMJ tenderness

Tenderness of TMJ was evaluated by the same method used in TMJ clicking. While LPM tenderness was assessed by intraoral palpation behind the tuberosity by the index finger. Both LPM and TMJ tenderness was recorded through visual analogue scale (VAS) with rating 0(no pain) and 10 (the severest pain).

### B) For group I (study), a 3D model of LPM and skeletal and dental structures and a needle insertion guide were designed for each patient as the following

**Fig.2:**
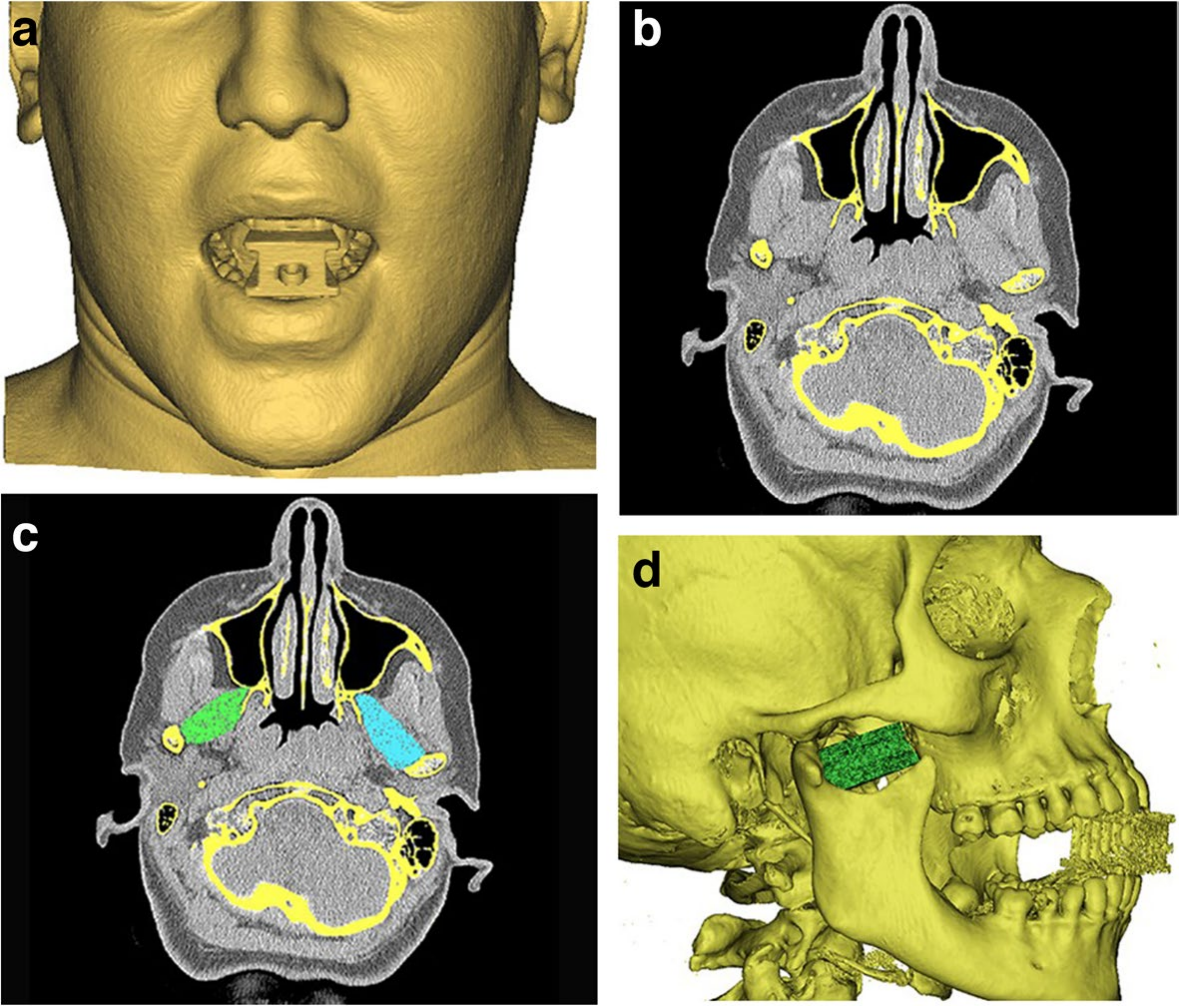
**a:** CT scan with soft tissue window on patient’s face while biting on a readymade bite block on the anterior teeth to prevent inter-arch teeth contact. **b:** Axial CT section showing right and left LPMs before segmentation**. c:** Right and left LPMs after segmentation in the same axial CT section **d:** 3D augmented model showing skull bone and teeth together with segmented LPM in its exact position

An intraoral digital scan for all the maxillary teeth using intraoral scanner (CEREC Omnicam AC; Dentsply Sirona) was performed and saved as standard tessellation language (STL) file. Then computed tomography (CT) scan with soft tissue window (slice width 0.5 mm) was done on patient’s face while biting on a readymade bite block on the anterior teeth to prevent inter-arch teeth contact and saved as Digital Imaging and Communications in Medicine (DICOM) format. (Fig. 2a).

The CT DICOM was then imported into Mimics software system (Mimics innovation suite; Materialise) and segmentation done to perform 3D model: first, segmentation of skull bone alone without any soft tissue involvement after confirmations from all the cuts. Second, segmentation of the right and left LPMs using lasso tool in all the axial cuts showing LPMs and merging of the segmented LPMs and the segmented skull bone together in one 3D model and exported as STL file. (Fig. 2b, c, d).

**Fig.3:**
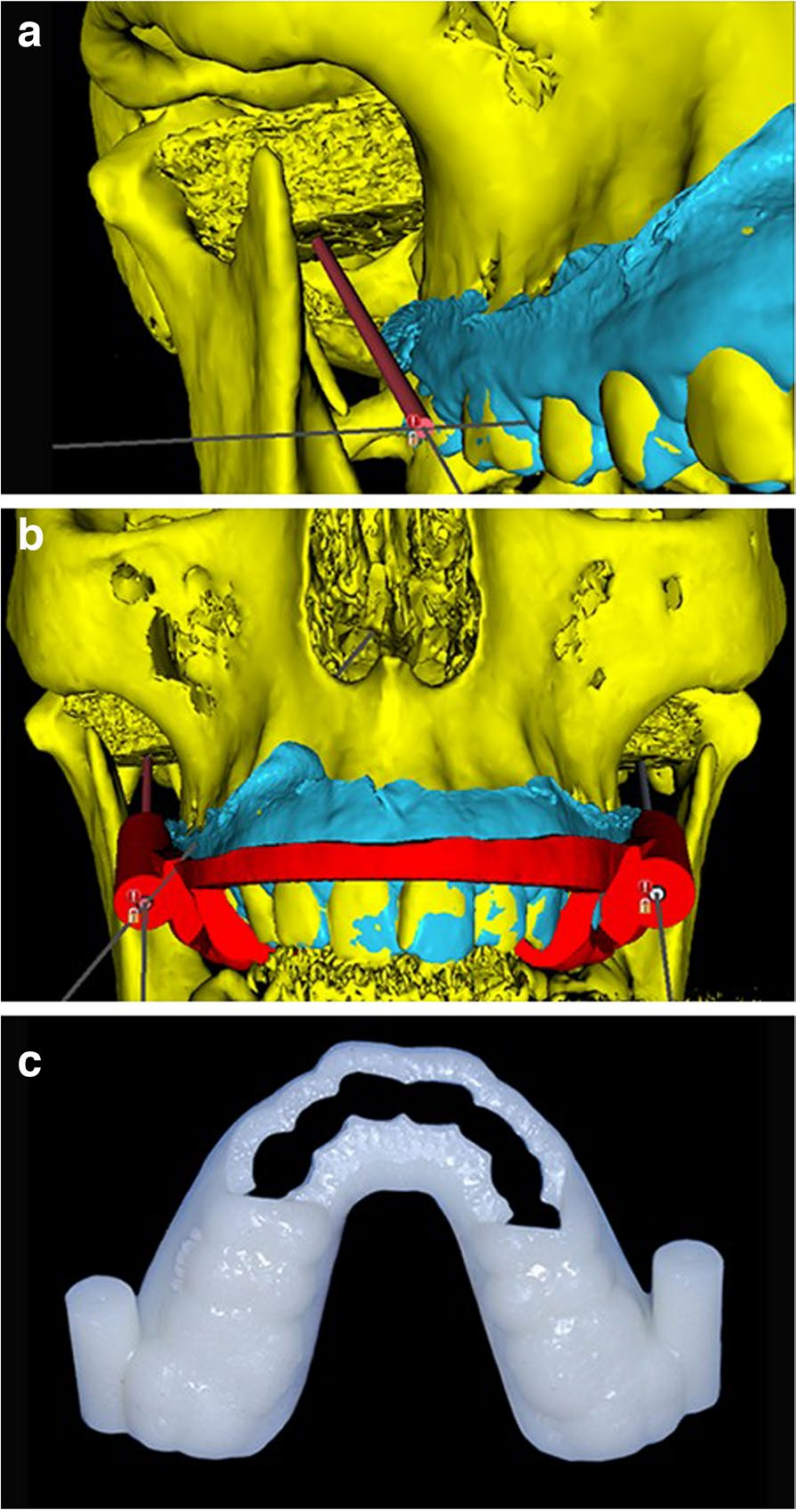
**a:** Planning the needle pathway and its insertion point in the LPM. **b:** The needle insertion guide resting on the 3D model with an anterior window to facilitate placement of bite block during injection. **c:** Customized needle insertion guide with two needle guide tubes

The CT DICOM and both STL files of upper jaw scan and 3D segmented model of LPMs and skull bone and dentition were imported into blue sky plan software (Blue sky plan V 4.7; Blue Sky Bio). A customized implant was used to represent the injection needle. Needle insertion guide with a guide tube (Hole diameter of 1.5 mm, from 15–20 mm height and zero offset) was designed after planning the needle direction and depth starting from the buccal surface of upper molars till the center of the muscle and needle position was confirmed from all views of 3D. Then, the guide was printed using manufactured resin (Esun standard resin; Esun). (Fig. 3a, b, c).

### C) Botulinum toxin injection for both groups

**Fig.4:**
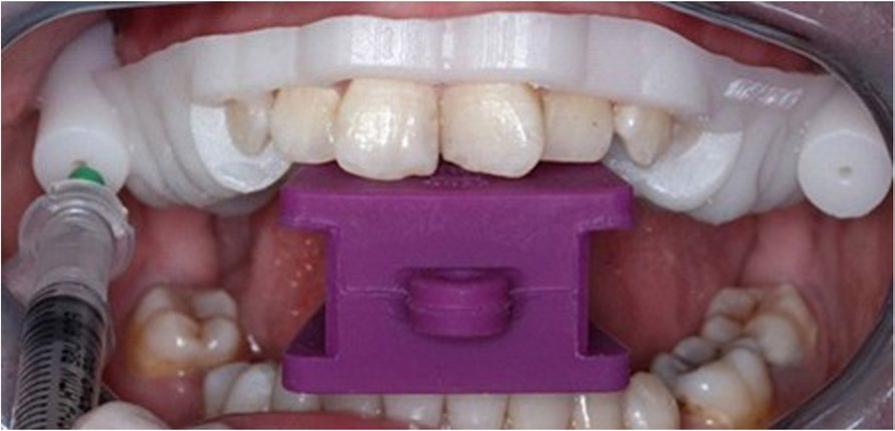
Showing needle insertion guide resting on upper teeth with the same readymade bite block used in CT scan during BTX injection

For group I, the patient was seated in upright position and his customized needle insertion guide was inserted and the patient was asked to bite on the same bite block used during CT scan. 25 units of BTX-A (Botox; Allergan) was prepared according to the manufacture instructions and then injected after confirming negative aspiration toward the symptomatic LPM using 22-gauge cannula needle prepared according to the planned length. Then, the patient was asked to manipulate the mandible. (Fig. 4).

**Fig.5:**
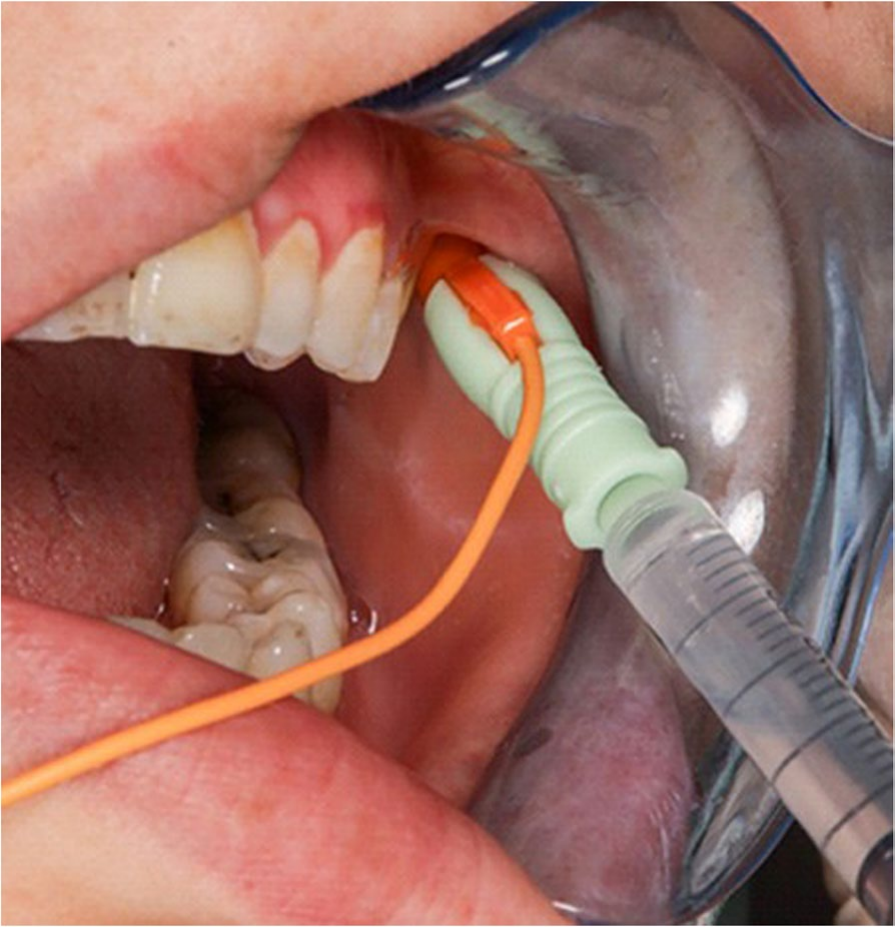
EMG needle inserted intraorally targeting LPM

For group II, 27-gauge monopolar cannulated needle electrode was inserted in the symptomatic LPM above the upper molar, lateral to the maxillary tuberosity [7, 9]. The patient was asked to manipulate the mandible till a loud sound was heard from electromyographic device (Neuro-EMG-Micro; Neurosoft) followed by aspiration and injection of 25 units of same BTX-A type. (Fig. 5).

### D) Postoperative instructions

Patients in both groups were asked to manipulate the mandible by opening and closing several times post injection and to stay in upright position for 4 h to avoid BTX-A diffusion into the surrounding pharyngeal muscles which may cause complications such as dysphagia and nasal regurgitation [11].

### Assessment

All patients were recalled at 1-,3- and 6-month post injection for evaluation of MIO, TMJ clicking and LPM and TMJ tenderness same as mentioned preoperatively and the articular disc position was evaluated at 3-month post injection.

#### For Articular disc position

**Fig.6:**
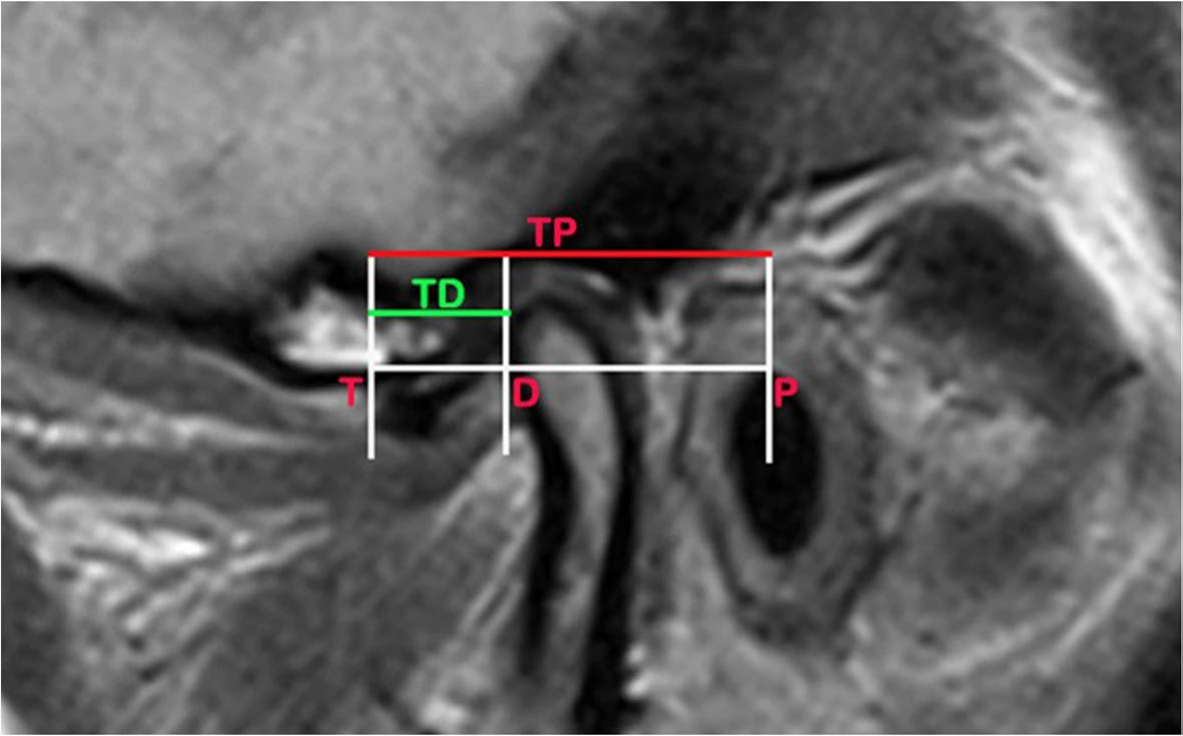
Measuring the relative disc position (TD/TP) using kruita et al. method

Disc position was monitored on MRI using Kruita et al. method [27] by drawing a tangent extending from the lowest edge of the articular eminence (T) to the highest edge of the external auditory canal (P). Then, point (D) was drawn at the intersection between line at the posterior part of the disc and the previous tangent. The relative disc position was calculated as TD/TP after measuring distance of TD and TP in millimeters. (Fig. 6).

### Sample size calculation

Based on previous studies [28, 29], sample size was calculated to be 20 patients (*n* = 10 per group) to detect an effect size derived from the mean difference in the percent of change of MIO, assuming 5% alpha error and 80% study power. This sample size was based on Rosner’s method [30] and calculated by G* Power (3.1.9.7) [31].

### Randomization and blinding

The 20 patients were randomly allocated into 2 equal groups, group I (study) and group II (control), using a computer-generated list of random numbers with Microsoft excel software (Microsoft Excel v 2109; Microsoft corporation). The allocation concealment was done using continuously numbered sealed envelopes and the assistant opened the envelopes to identify the group to which the patient was allocated 2 days before the intervention to give time for guide fabrication for group 1. Only the outcome assessor and the data analyst were blinded as blinding the operator and the patients was not possible.

### Statistical analysis

Data were fed to the computer and analyzed using IBM SPSS software package version 20.0 (Armonk, NY: IBM Coro). Qualitative data were described using number and percent. The Shapiro–Wilk test was used to verify the normality of distribution. Significance of the obtained results was judged at the 5% level. For categorical variables, different groups were compared using either Chi-square test or Fisher’s Exact. For normally distributed quantitative data, two studied groups were compared by Student t-test and ANOVA with repeated measures was used to compare between more than two periods and Post Hoc Test (adjusted Bonferroni) for pairwise comparisons. For abnormally distributed quantitative variables, Mann Whitney test was used to compare between the two studied groups and Friedman test was used to compare between more than two periods and Post Hoc Test (Dunn's) for pairwise comparisons.

## Results

### Participants’ baseline data and flow

Twenty patients were enrolled in this clinical trial based on the previously mentioned inclusion and exclusion criteria without any drop out in either group. Group I included nine females and one male while group II included ten females. The mean age of group I was (27.30 ± 5.36) years while (23.10 ± 2.85) years for group II.

### Outcomes

#### Maximum interincisal opening

Throughout the study follow-up period, there was no statistically significant difference between both groups. (*p* = 0.201, 0.122, 0.080 respectively).

**Fig.7:**
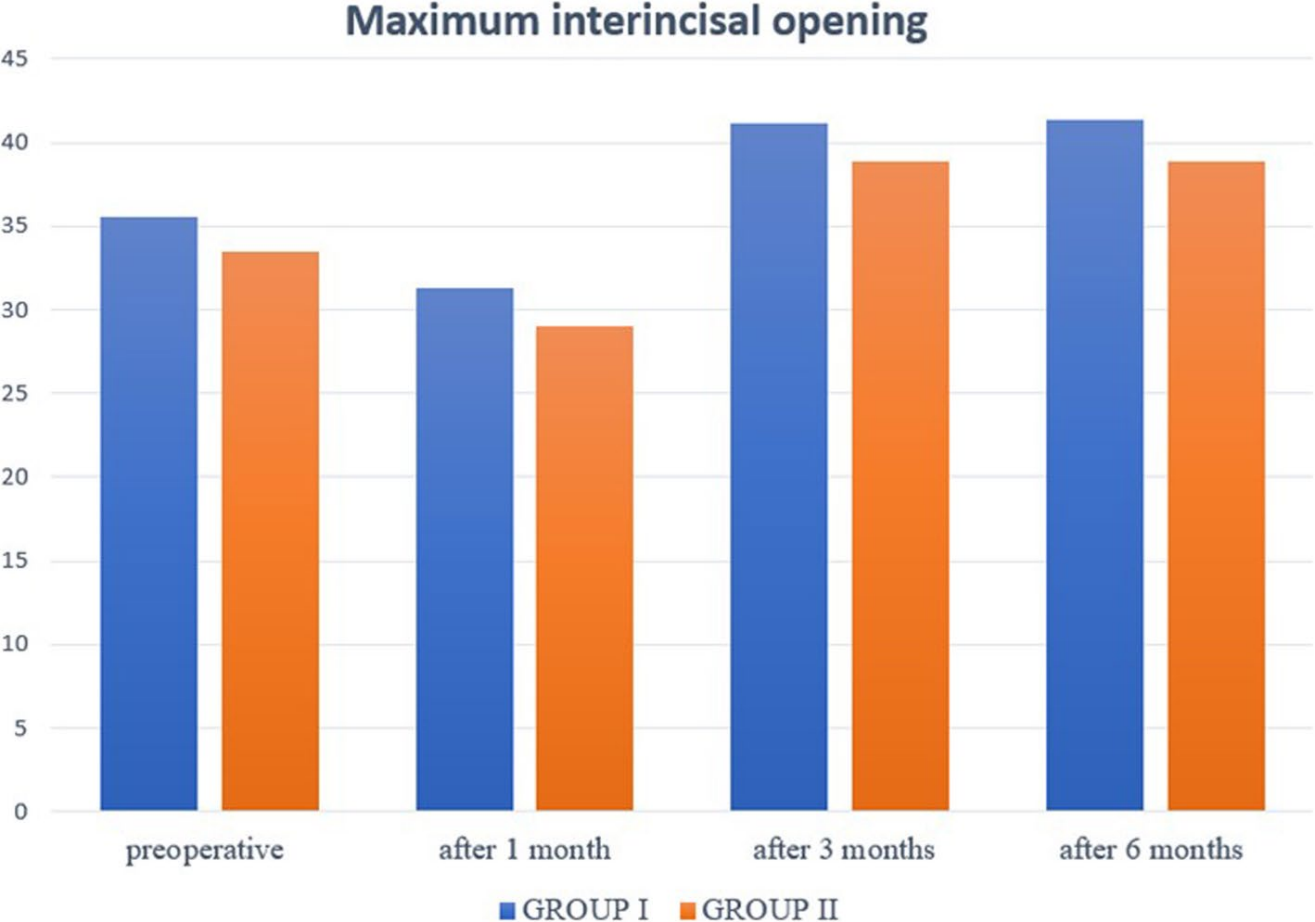
Bar chart showing mean of maximum interincisal opening in millimeters during follow-up periods

Maximum interincisal opening decreased insignificantly in group I (*p* = 0.163) and significantly in group II (*p* < 0.001^*^) at 1-month follow-up. While at 3-and 6-month follow-up, MIO increased significantly in both groups (*p* < 0.001^*^). (Fig. 7).

#### Measurement of articular disc position

**Fig.8:**
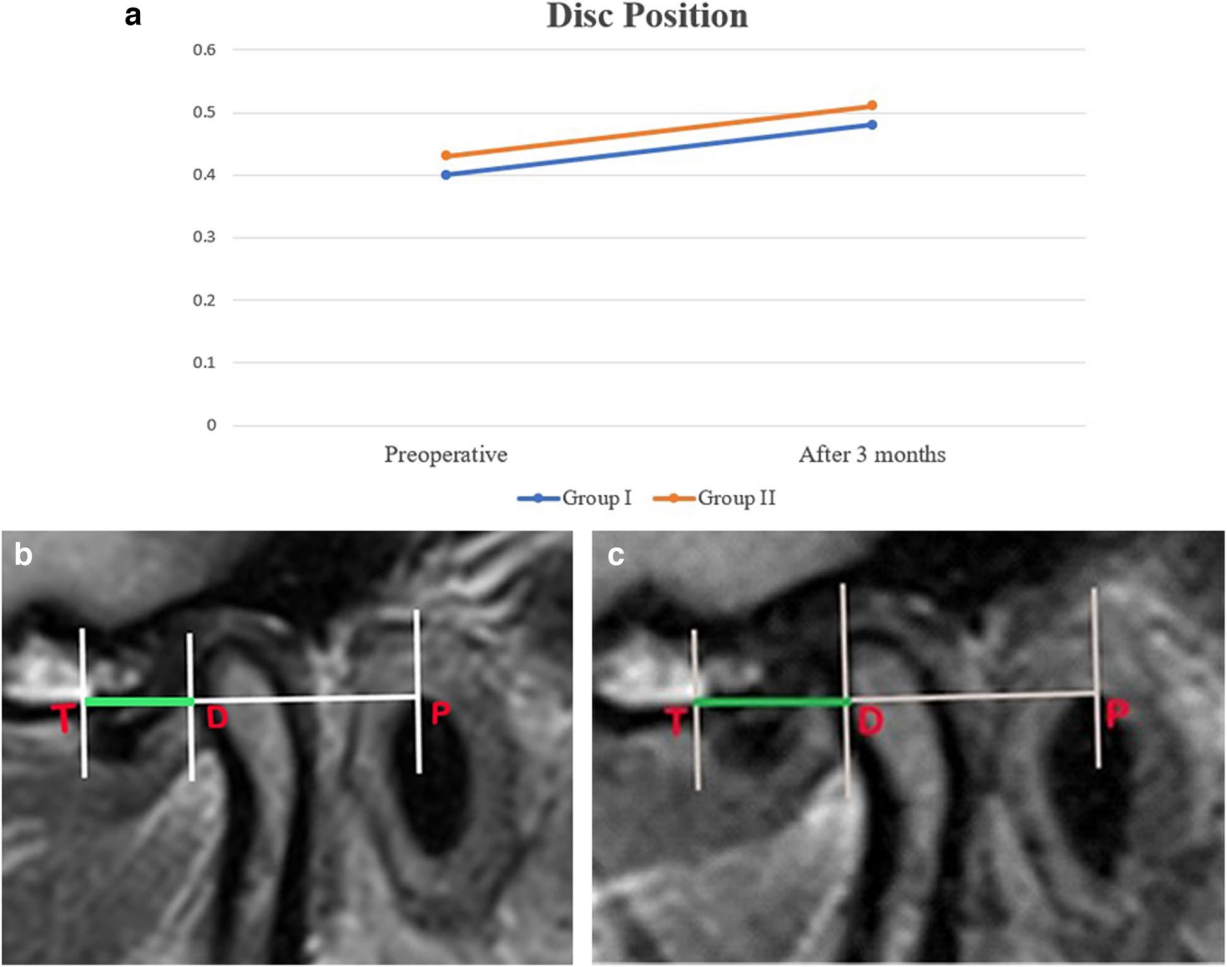
**a:** Line graph showing increase mean of TD/TP in both groups at 3-month follow-up. **b, c:** Pre-and post-operative MRI showing reduction and improvement of the articular disc position

A statistically significant increase in mean of TD/TP from 0.40 ± 0.04 and 0.43 ± 0.03 respectively to be 0.48 ± 0.04 for group I and 0.51 ± 0.05 for group II. Despite that, there was no statistically significant difference between both groups at the evaluation time (*p* = 0.291). (Fig. 8 a, b, c).

#### Clicking

**Fig.9:**
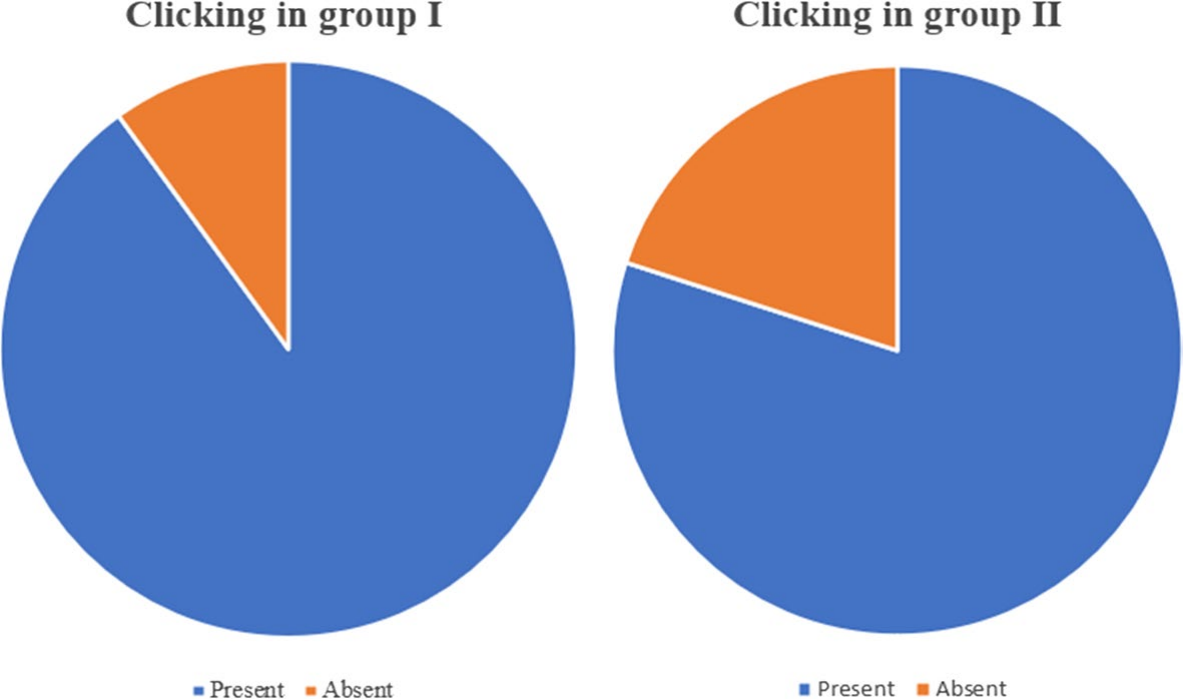
**a, b:** Pie chart showing presence and absence of clicking at 3- and 6-month follow-up in group I and II

In both groups, all patients (*n* = 20) had no clicking after 1 month; however, one patient in group I and two patients in group II regained clicking at 3-month follow-up. (Fig. 9a, b).

#### Lateral pterygoid muscle tenderness

Both groups showed statistically significant reduction in LPM tenderness throughout the study period. However, the difference between both groups was not statistically significant except at 3-and 6-month follow-up were in favor of group I. (Table 1).

**Table 1:** Inter and intragroup comparisons according to Lateral pterygoid muscle tenderness

Lateral pterygoid muscle tenderness	Test (*n* = 10)	Control (*n* = 10)	Test statistic	*p*-value
**Pre-operative**				
Mean ± SD	9.20 ± 1.75	8.90 ± 1.73	42.0	0.579
Median (IQR)	10.0^a^ (10.0–10.0)	10.0^a^ (8.0–10.0)		
**After 1 month**				
Mean ± SD	0.30 ± 0.95	1.70 ± 1.83	24.50	0.052
Median (IQR)	0.0^b^ (0.0–0.0)	1.0^c^ (0.0–4.0)		
**After 3 months**				
Mean ± SD	0.70 ± 1.34	3.60 ± 2.37	16.50^*^	0.009^*^
Median (IQR)	0.0^b^ (0.0–1.0)	4.0^bc^ (2.0–5.0)		
**After 6 months**				
Mean ± SD	1.10 ± 1.91	3.70 ± 2.21	18.00^*^	0.015^*^
Median (IQR)	0.0^b^ (0.0–2.0)	4.0^b^ (2.0–5.0)		
**Test statistic**	26.125^*^	27.536^*^		
**p**_**1**_	< 0.001^*^	< 0.001^*^		

*IQR* Inter quartile range, *SD* Standard deviation, Values with totally Different letters ^(a−c)^ are significant,

^*^Statistically significant at *p* ≤ 0.05

#### Temporomandibular joint tenderness

Both groups showed statistically significant reduction in TMJ tenderness at 1-,3-and 6-month follow-up. However, the difference in TMJ tenderness reduction was not statistically significant between both groups at 1,3- and 6-month follow-up (*p* = 1, = 0.739 and = 0.739 respectively).

## Discussion

Botulinum toxin Injection in LPM is considered an effective minimally invasive treatment modality for DDWR as well as chronic TMJ dislocation and oromandibular dystonia [5, 9, 11, 21, 32, 33].Due to the deep location, complicated surrounding anatomy of LPM and variation among individuals, guidance techniques such as EMG, ultrasonography and MRI guided navigation, being the most commonly introduced, are mandatory [20–22, 29].Electromyography is a useful modality for muscle detection and the most commonly mentioned in literature, however, it is limited by the operator skills for the correct needle insertion into LPM as it considers only functional guidance depending on signals from contracting muscles not an actual visualization of full dimension of the muscle [20, 21].As a result of that, EMG does not prevent out target injection for BTX into adjacent muscles leading to dysphagia, nasal regurgitation, nasal tone involvement and flu like symptoms in some reported cases undergoing injection under EMG [11, 34, 35]. For ultrasonography, although it is a real time image for guided LPM injection, it is not reproducible due to surrounding bony structures around LPM hindering its easily performance by clinicians [20, 22, 36].While for MRI guided navigation, the need for clinician experience and MRI navigation system hinder its performance on daily dental practice [20, 29].All of the previously mentioned guidance techniques require either the experience or special equipment to allow their performance in dental clinic and on repeated settings.

In the current study, a technique of fully guided BTX injection in LPM using muscle segmentation technique for LPM based on CT with soft tissue window, being a reliable method of viewing and measuring of actual muscle mass, is introduced [37]. In this technique, the LPM appeared in its actual dimension and its full depth together with a 3D virtual augmented model that mimics patient dental and skeletal structures. After that, virtual planning was started to determine needle direction, depth and insertion site in the center of the muscle followed by fabrication of needle insertion guide using computer aided design/computer aided manufacture (CAD/CAM). Needle insertion guides mentioned in literature, unlike the current study, were fabricated based on estimation of the muscle’s location according to its attachment using CT or cone-beam computed tomography (CBCT) only without true visualization of the muscle. Therefore, those guides necessitate the use of EMG to confirm needle insertion into LPM [21, 38].

This clinical trial, according to the authors’ knowledge, is the first clinical trial to compare between a newly introduced guidance technique for BTX injection in LPM and the standard guidance technique for managing DDWR patients.

Initial diagnosis of DDWR was based on patient’s history and clinical examination followed by MRI confirmation according to DC/TMD [1]. In this clinical trial, injection was performed in the center of the muscle without differentiation between superior and inferior heads of LPM depending on previous studies which consider all heads of LPM as one single muscular system [5, 9, 39].

In the present study, MIO dropped insignificantly in group I while significantly in group II at 1-month follow-up. However, that was followed by improvement in MIO in both groups at 3- and 6-month follow-up with no statistical difference between them. This MIO improvement agrees with Pons et al. who mentioned significant MIO improvement with MRI guided navigation and Altaweel et al. who detected reduction till 2 months postoperatively and followed by improvement at 3-,4-and 6-month follow-up using EMG [20, 40]. While our result is contradictory to Emara et al. who showed reduction in MIO, though it was not significant at 4-month post injection [11].

Regarding TMJ clicking, all patients lost clicking at 1-month follow-up in both groups. However, only one patient regained clicking in group I and two patients regained clicking in group II at 3-month follow-up and till the end of the study without any further injection. This result comes in accordance with Emara et al. and Taema et al. using EMG who stated that clicking reappearance in some patients can be explained by fading the effect of BTX-A [9, 11].

For disc position, both guidance techniques showed significant improvement in disc position with no statistical difference between 2 groups and this agrees with the results of Taema et al. and Emara et al. who explained that improvement by the theory explaining the role of LPM hyperactivity in counteracting retrodiscal tissue action to displace the articular disc anteriorly [9, 11, 41]. Reduction in TMJ tenderness was statistically significant in both groups at 1-,3-and 6-month follow-up with no statistically significant difference between both groups. For LPM tenderness, reduction was detected significantly in both groups. This result conforms with Altaweel et al. who documented reduction in TMJ and LPM tenderness after BTX injection using EMG [40]. However, the difference between both groups was not statistically significant at 1-month follow-up and statistically significant at 3-and 6-month follow-up in favor of group I in LPM tenderness reduction.

Relief of symptoms such as clicking, LPM and TMJ tenderness can be explained by the pain inhibition and paralyzing effect of BTX at the 1-and 3-month follow-up [40]. While prolonged effect of these symptoms can be justified by reduction of muscle hyperactivity even after fading effect of BTX due to deprogramming of the muscle [34, 40].

By the end of this study, 3D guided BTX injection in LPM using muscle segmentation technique showed no statistically significant difference with the result of EMG, the most commonly applied guidance technique, except for reduction of LPM tenderness by VAS where the result was in favor of the 3D guided technique.

Limitations of this technique include skills required for utilizing the planning software for muscle segmentation which necessitates dedicated training time and a learning curve for the clinician and the CT radiation dose exposure. However, each patient will perform CT once for fabricating the guide to use it whenever needed on the dental chair in subsequent settings as repeatable injection after fading of BTX effect might be needed in different TMDs as mentioned by other studies [7]. Therefore, the cost- effectiveness ratio is in favour of 3D guidance modality, which involves only the cost of CT with soft tissue window and the cost of guide fabrication, compared to the EMG guidance modality, which includes the cost of EMG device and the cost of disposable EMG needle that will be required in each repeated LPM injection for the same patient.

The current study has introduced manual muscle segmentation for LPM based on CT with soft tissue window which is considered a reliable and accurate modality for muscle mass assessment. As the validity of CT in muscle identification and segmentation has been assessed in several studies using whether manual or semiautomated techniques [42, 43]. The relatively short follow-up period of our study is considered a limitation. So, we recommend future studies with a longer follow up. Additionally, comparing 3D guidance modality with other guidance modalities for LPM injection is recommended.

With recent development in augmented reality and artificial intelligence technology, future studies are recommended to validate the feasibility of CBCT in LPM segmentation owing to its availability and its lower dose of radiation. Furthermore, the introduced technique of 3D guidance modality and LPM segmentation can be applied for other hyperactive masticatory muscles as medial pterygoid muscle. The 3D guidance modality for LPM can also be effective in other conditions affecting oral and maxillofacial region and related to muscle hyperactivity, such as chronic TMJ dislocation, oromandibular dystonia and condylar fracture management.

## Conclusion

The findings of this study suggest that the fully guided technique using muscle segmentation is a viable, cost-effective and reproducible alternative to EMG for BTX injection in LPM.

## Data Availability

The data of this study are available with the corresponding author.

## Abbreviations

BTX: Botulinum toxin
LPM: Lateral pterygoid muscle
DDWR: Disc displacement with reduction
TMJ: Temporomandibular joint
EMG: Electromyography
RCT: Randomized controlled trial
DDWoR: Disc displacement without reduction
DC/TMD: Diagnostic criteria of temporomandibular disorders
BTX-A: Botulinum toxin type A
CT: Computed tomography
CBCT: Cone-beam computed tomography
STL: Standard tessellation language file
DICOM: Digital imaging and communications in medicine format
MIO: Maximum interincisal opening
VAS: Visual analogue scale
CAD/CAM: Computer aided design/ computer aided manufacture

## References

[1] Schiffman E, Ohrbach R, Truelove E, Look J, Anderson G, Goulet J-P, et al. Diagnostic Criteria for Temporomandibular Disorders (DC/TMD) for clinical and research applications: Recommendations of the international RDC/TMD consortium network* and orofacial pain special interest group. J Oral Facial Pain Headache. 2014;28:6–27. 24482784 10.11607/jop.1151 PMC4478082

[2] Rongo R, Ekberg E, Nilsson I-M, Al-Khotani A, Alstergren P, Conti PCR, et al. Diagnostic criteria for temporomandibular disorders (DC/TMD) for children and adolescents: An international Delphi study-Part 1-Development of Axis I. J Oral Rehabil. 2021;48:836–45. 10.1111/joor.13175. 33817818 10.1111/joor.13175 PMC8252391

[3] Valesan LF, Da-Cas CD, Réus JC, Denardin ACS, Garanhani RR, Bonotto D, et al. Prevalence of temporomandibular joint disorders: a systematic review and meta-analysis. Clin Oral Investig. 2021;25:441–53. 33409693 10.1007/s00784-020-03710-w

[4] Elfving L, Helkimo M, Magnusson T. Prevalence of different temporomandibular joint sounds, with emphasis on disc-displacement, in patients with temporomandibular disorders and controls. Swed Dent J. 2002;26:9–19. 12090160

[5] Rady NA, Bahgat MM, Abdel-Hamid AM. Promising minimally invasive treatment modalities for symptomatic temporomandibular joint disc displacement with reduction: a randomized controlled clinical trial. BMC Oral Health. 2022;22:547. 36456937 10.1186/s12903-022-02579-3 PMC9714147

[6] Marpaung C, van Selms MKA, Lobbezoo F. Temporomandibular joint anterior disc displacement with reduction in a young population: Prevalence and risk indicators. Int J Paediatr Dent. 2019;29:66–73. 30218477 10.1111/ipd.12426

[7] Bakke M, Møller E, Werdelin LM, Dalager T, Kitai N, Kreiborg S. Treatment of severe temporomandibular joint clicking with botulinum toxin in the lateral pterygoid muscle in two cases of anterior disc displacement. Oral Surg Oral Med Oral Pathol Oral Radiol Endod. 2005;100:693–700. 10.1016/j.tripleo.2004.11.019. 16301150 10.1016/j.tripleo.2004.11.019

[8] Patel K, Eley KA, Cascarini L, Watt-Smith S, Larkin M, Lloyd T, et al. Temporomandibular disorders-review of evidence-based management and a proposed multidisciplinary care pathway. Oral Surg Oral Med Oral Pathol Oral Radiol. 2023;136:54–69. 36990844 10.1016/j.oooo.2023.02.001

[9] Taema M, Nabi NA, Ibrahim S, Kamal HA, Emara A. Assessment of anterior positioning splint in conjunction with lateral pterygoid BTX injection to treat TMJ disc displacement with reduction - a preliminary report. Maxillofac Plast Reconstr Surg. 2021;43:33. 34495418 10.1186/s40902-021-00317-3 PMC8426453

[10] Song PC, Schwartz J, Blitzer A. The emerging role of botulinum toxin in the treatment of temporomandibular disorders. Oral Dis. 2007;13:253–60. 17448205 10.1111/j.1601-0825.2007.01352.x

[11] Emara AS, Faramawey MI, Hassaan MA, Hakam MM. Botulinum toxin injection for management of temporomandibular joint clicking. Int J Oral Maxillofac Surg. 2013;42:759–64. 23538215 10.1016/j.ijom.2013.02.009

[12] Sato S, Goto S, Nasu F, Motegi K. Natural course of disc displacement with reduction of the temporomandibular joint: changes in clinical signs and symptoms. J Oral Maxillofac Surg. 2003;61:32–4. 12524604 10.1053/joms.2003.50005

[13] Ghoneim NI, Mansour NA, Elmaghraby SA, Abdelsameaa SE. Treatment of temporomandibular joint disc displacement using arthrocentesis combined with injectable platelet rich fibrin versus arthrocentesis alone. J Dent Sci. 2022;17:468–75. 10.1016/j.jds.2021.07.027. 35028072 10.1016/j.jds.2021.07.027 PMC8739728

[14] Castaño-Joaqui OG, Cano-Sánchez J, Campo-Trapero J, Muñoz-Guerra MF. TMJ arthroscopy with hyaluronic acid: A 12-month randomized clinical trial. Oral Dis. 2021;27:301–11. 32609918 10.1111/odi.13524

[15] Aoki KR, Guyer B. Botulinum toxin type A and other botulinum toxin serotypes: a comparative review of biochemical and pharmacological actions. Eur J Neurol. 2001;8:21–9. 11851731 10.1046/j.1468-1331.2001.00035.x

[16] Shabaan AA, Kassem I, Aboulmagd I, Amer IA, Shaaban A, Abd-El-Ghafour M, et al. Effectiveness of intra-oral botulinum toxin injection in comparison to the extra-oral approach on pain and quality of life in patients with myofascial pain: a randomized clinical trial. Clin Oral Investig. 2024;29:18. 10.1007/s00784-024-06051-0. 39681752 10.1007/s00784-024-06051-0 PMC11649703

[17] Kim Y-M, Son J-Y, Ahn D-K. Botulinum toxin type A is a potential therapeutic drug for chronic orofacial pain. J Oral Biosci. 2024;66:496–503. 38908515 10.1016/j.job.2024.06.004

[18] Rahmatipour H, Shabestari SM, Benisi SZ, Samadikhah H. Pioneering pain management with botulinum toxin type A: From anti-inflammation to regenerative therapies. Heliyon. 2025;11:e42350. 40028584 10.1016/j.heliyon.2025.e42350 PMC11870196

[19] Stöckle M, Fanghänel J, Knüttel H, Alamanos C, Behr M. The morphological variations of the lateral pterygoid muscle: A systematic review. Ann Anat. 2019;222:79–87. 30394300 10.1016/j.aanat.2018.10.006

[20] Pons M, Meyer C, Euvrard E, Weber E, Sigaux N, Louvrier A. MR-guided navigation for botulinum toxin injection in the lateral pterygoid muscle. First results in the treatment of temporomandibular joint disorders. J Stomatol Oral Maxillofac Surg. 2019;120:188–95.10.1016/j.jormas.2018.11.002 10.1016/j.jormas.2018.11.002 30453102

[21] Yoshida K. Computer-aided design/computer-assisted manufacture-derived needle guide for injection of botulinum toxin into the lateral pterygoid muscle in patients with oromandibular dystonia. J Oral Facial Pain Headache. 2018;32:e13-21. 29694466 10.11607/ofph.1955

[22] Lee S-T, Kim D, Park J-H, Kwon T-G. Ultrasound-guided intraoral botulinum toxin injection into the lateral pterygoid muscle for chronic temporomandibular joint dislocation. J Korean Assoc Oral Maxillofac Surg. 2024;50:41–8. 38419520 10.5125/jkaoms.2024.50.1.41 PMC10910006

[23] Ayoub A, Pulijala Y. The application of virtual reality and augmented reality in Oral & Maxillofacial Surgery. BMC Oral Health. 2019;19:238. 10.1186/s12903-019-0937-8. 31703708 10.1186/s12903-019-0937-8 PMC6839223

[24] Kwon H-B, Park Y-S, Han J-S. Augmented reality in dentistry: a current perspective. Acta Odontol Scand. 2018;76:497–503. 29465283 10.1080/00016357.2018.1441437

[25] Schulz KF, Altman DG, Moher D. CONSORT 2010 statement: Updated guidelines for reporting parallel group randomised trials. J Pharmacol Pharmacother. 2010;1:100–7. 21350618 10.4103/0976-500X.72352 PMC3043330

[26] General Assembly of the World Medical Association. World Medical Association Declaration of Helsinki: ethical principles for medical research involving human subjects. J Am Coll Dent. 2014;81:14–8. 25951678

[27] Kurita H, Kurashina K, Ohtsuka A, Kotani A. Change of position of the temporomandibular joint disk with insertion of a disk-repositioning appliance. Oral Surg Oral Med Oral Pathol Oral Radiol Endod. 1998;85:142–5. 9503446 10.1016/s1079-2104(98)90416-4

[28] Montes-Carmona J-F, Gonzalez-Perez L-M, Infante-Cossio P. Treatment of localized and referred masticatory myofascial pain with botulinum toxin injection. Toxins (Basel). 2020;13:6. 33374687 10.3390/toxins13010006 PMC7822413

[29] Martenot A, Devoti JF, Pons M, Meyer C, Brumpt E, Louvrier A, et al. Persistent myogenic temporomandibular disorders: Are navigation-guided botulinum toxin-A injections into the lateral pterygoid muscles effective? J Stomatol Oral Maxillofac Surg. 2024;125:101715. 38013116 10.1016/j.jormas.2023.101715

[30] Rosner B. Sample inference. In: Fundamentals of Biostatistics. 7th ed. Boston: Brooks/Cole, Cengage Learning; 2015. p. 269-301

[31] Universität Düsseldorf. G*Power.2019. http://www.gpower.hhu.de/

[32] Tocaciu S, McCullough MJ, Dimitroulis G. Surgical management of recurrent TMJ dislocation-a systematic review. Oral Maxillofac Surg. 2019;23:35–45. 10.1007/s10006-019-00746-5. 30729355 10.1007/s10006-019-00746-5

[33] Martínez-Pérez D, García R-E. Recurrent temporomandibular joint dislocation treated with botulinum toxin: report of 3 cases. J Oral Maxillofac Surg. 2004;62:244–6. 10.1016/j.joms.2003.04.014. 14762760 10.1016/j.joms.2003.04.014

[34] Hassan M, Emara A, Hakam M, Elfarmawy M. MRI Monitoring of Disc Position Changes Following Botulinum Toxin Injection for Management of TMJ Clicking. Med J Cairo Univ. 2013;81:11–20.

[35] Kanbour A, Hurrell MJL, Ricciardo P. Velopharyngeal dysfunction following botulinum toxin type A injection to the lateral pterygoid muscles for recurrent jaw dislocation. BMJ Case Rep. 2021;14:e238766. 10.1136/bcr-2020-238766 PMC8070878 33888473

[36] Rodríguez-Gimillo P, Valverde-Navarro A, Margaix-Muñoz M, Poveda-Roda R, Delgado-Navarro C, Puig-Bernabeu J. Lateral pterygoid muscle ultrasound-guided injection: A technical note. J Stomatol Oral Maxillofac Surg. 2024;125:101547. 37394100 10.1016/j.jormas.2023.101547

[37] Yao N, Li X, Wang L, Cheng X, Yu A, Li C, et al. Deep learning for automatic segmentation of paraspinal muscle on computed tomography. Acta Radiol. 2023;64:596–604. 35354336 10.1177/02841851221090594

[38] Casatuto T, Gosselin M, Lerhe B, Vandersteen C, Ehrmann E, Savoldelli C. In-house tooth-supported guide for the injection of botulinum toxin into the lateral pterygoid muscle using Blue Sky Plan software: A technical note. J Stomatol Oral Maxillofac Surg. 2021;122:e77-80. 34157446 10.1016/j.jormas.2021.05.015

[39] Murray GM, Bhutada M, Peck CC, Phanachet I, Sae-Lee D, Whittle T. The human lateral pterygoid muscle. Arch Oral Biol. 2007;52:377–80. 17141177 10.1016/j.archoralbio.2006.10.002

[40] Altaweel AA, Elsayed SAH, Baiomy AABA, Abdelsadek SE, Hyder AA. Extraoral versus intraoral botulinum toxin type A injection for management of temporomandibular joint disc displacement with reduction. J Craniofac Surg. 2019;30:2149–53. 31232992 10.1097/SCS.0000000000005658

[41] Litko M, Szkutnik J, Berger M, Różyło-Kalinowska I. Correlation between the lateral pterygoid muscle attachment type and temporomandibular joint disc position in magnetic resonance imaging. Dentomaxillofac Radiol. 2016;45:20160229. 27506381 10.1259/dmfr.20160229 PMC5595028

[42] Graffy PM, Liu J, Pickhardt PJ, Burns JE, Yao J, Summers RM. Deep learning-based muscle segmentation and quantification at abdominal CT: application to a longitudinal adult screening cohort for sarcopenia assessment. Br J Radial. 2019;92:20190327. 10.1259/bjr.20190327 PMC6724622 31199670

[43] Park HJ, Shin Y, Park J, Kim H, Lee IS, Seo DW, et al. Development and Validation of a Deep Learning System for Segmentation of Abdominal Muscle and Fat on Computed Tomography. Korean I Radiol. 2020;21:88–100. 10.3348/kjr.2019.0470 PMC6960305 31920032

